# Varied sensitivity to therapy of HIV-1 strains in CD4^+ ^lymphocyte sub-populations upon ART initiation

**DOI:** 10.1186/1742-6405-7-42

**Published:** 2010-12-06

**Authors:** Edwin J Heeregrave, Mark J Geels, Elly Baan, Renee M van der Sluis, William A Paxton, Georgios Pollakis

**Affiliations:** 1 Laboratory of Experimental Virology, Department of Medical Microbiology, Center of Infection and Immunity Amsterdam (CINIMA), Academic Medical Center of the University of Amsterdam, The Netherlands; 2 Mark J Geels is currently employed at Nobilon International BV, Exportstraat 39B, P.O. Box 320, 5830 AH Boxmeer, The Netherlands

## Abstract

**Background:**

Although antiretroviral therapy (ART) has proven its success against HIV-1, the long lifespan of infected cells and viral latency prevent eradication. In this study we analyzed the sensitivity to ART of HIV-1 strains in naïve, central memory and effector memory CD4^+ ^lymphocyte subsets.

**Methods:**

From five patients cellular HIV-1 infection levels were quantified before and after initiation of therapy (2-5 weeks). Through sequencing the C2V3 region of the HIV-1 gp120 envelope, we studied the effect of short-term therapy on virus variants derived from naïve, central memory and effector memory CD4^+ ^lymphocyte subsets.

**Results:**

During short-term ART, HIV-1 infection levels declined in all lymphocyte subsets but not as much as RNA levels in serum. Virus diversity in the naïve and central memory lymphocyte populations remained unchanged, whilst diversity decreased in serum and the effector memory lymphocytes. ART differentially affected the virus populations co-circulating in one individual harboring a dual HIV-1 infection. Changes in V3 charge were found in all individuals after ART initiation with increases within the effector memory subset and decreases found in the naïve cell population.

**Conclusions:**

During early ART virus diversity is affected mainly in the serum and effector memory cell compartments. Differential alterations in V3 charge were observed between effector memory and naïve populations. While certain cell populations can be targeted preferentially during early ART, some virus strains demonstrate varied sensitivity to therapy, as shown from studying two strains within a dual HIV-1 infected individual.

## Background

Antiretroviral therapy (ART) has proven to be successful against human immunodeficiency virus type 1 (HIV-1) and results in undetectable plasma levels for many years. However, an increasing number of studies report on adverse events and toxicities [1,2]. Additional drawbacks to therapy are adherence and the considerable costs. In certain situations a more simplified antiretroviral regimen may be suitable, for instance as short-term use to prevent mother-to-child-transmission (MTCT), maintenance therapy after HAART or possibly as pre-exposure prophylaxis [3-7]. Despite the increased likelihood of viremia and emergence of resistance, prophylactic and/or short-term therapeutic use largely bypasses these disadvantages and more treatment options remain available.

The CD4^+ ^lymphocyte is the main target cell for HIV-1 infection with the various sub-populations infected to a different extent [8,9]. Naïve and memory lymphocyte subsets differ in body distribution, proliferative capacity and in expression levels of the main co-receptors for HIV-1, CCR5 and CXCR4 [10-13]. Despite these differences, all cellular subsets are productively infected and display a lack of viral compartmentalization among circulating cells in peripheral blood [9,14,15]. Under the influence of long-term ART most studies describe a lack of viral compartmentalization among HIV-1 infected CD4^+ ^lymphocyte subsets [16-19]. Both central and transitional memory CD4^+ ^lymphocytes are regarded as cellular reservoirs for HIV-1 under therapy [20]. Baldanti and colleagues show that naïve and memory cell numbers and HIV-1 infection levels do not differ greatly from each other during therapy [21]. These studies focus mainly on long-term ART and do not describe the influence on the cell subset-specific quasi-species during early therapy intervention. We studied alterations to HIV-1 infection levels and viral diversity within specific cellular subsets after short-term ART.

## Methods

Five chronically HIV-1 infected individuals, who visited frequently the outpatient clinic of the Academic Medical Center (AMC) of the University of Amsterdam, the Netherlands, participated in this study. These patients received various antiviral regimens (Table 1) and their characteristics have been described previously [9]. Serum and peripheral blood mononuclear cells (PBMC) were obtained and frozen according to standard protocols. Viral loads were determined with the Versant HIV-1 RNA Assay (bDNA; Bayer Diagnostics, Leverkusen, Germany). Determination of HIV-1 subtype was performed by phylogenetic analyses and by blasting the sequences using the Los Alamos database [22]. This study was approved by the Medical Ethical Committee of the AMC and informed consent was provided by all participants.

**Table 1:** Patient characteristics

Patient	*Env*	therapy	# days	viral load (copies/ml)		CD4 count (cells/μl)	
	subtype	regimen	on ART	ART-	ART+	ART-	ART+
M11306	C	amprenavir	14	52,436	3,160	90	n.d.^b^
M12020	D	zidovudine	18	5,352	304	190	220
M12259	F	zidovudine	33	246,572	25,588	360	500
M13408	A	d4t, 3tc, rtv^a^	28	65,262	247	620	840
M16394	C	zidovudine	28	1,026	607	800	690

^a^d4t - stavudine, 3tc - lamivudine, rtv - ritonavir

^b ^not determined

PBMC were thawed and FACS-sorted as published previously [9]. Cells were stained with various antibodies and three CD4^+ ^lymphocyte subsets were sorted: naïve, CD57^- ^memory (or central memory) and CD57^+ ^memory (or effector memory) CD4^+ ^lymphocytes. All cell sorts were performed utilizing a modified FACS DIVA. Viral DNA from the cell subsets was isolated utilizing a silica-based method, which was also used for RNA isolation from serum [23]. Cellular HIV-1 infection levels were quantified using a semi-nested real-time PCR assay [9]. This assay targets the LTR segment of the virus genome where the second strand transfer takes place and quantifies only fully reverse transcribed HIV-1 genomic DNA and has high specificity for all major HIV-1 subtypes. We excluded HIV-1 quantifications of the naïve subset of patient M16394 before therapy as well as the effector memory subsets before and after therapy and the memory subset after therapy of patient M12259, since either the input (cell number or virus copies) was too low or the outcome was unreliable. AMV-RT (Madison, WI, USA) was used for reverse transcription of the serum-derived RNA. The C2V3 region (HXB2 nucleotide positions 7032-7301) of the HIV-1 envelope gene was amplified using AmpliTaq DNA polymerase (PE Applied Biosystem, Foster City, CA, USA). The primers (100 ng/μl) for the first-round PCR were 5'-AATGTCAGCACAGTACAATG-3' and 3'-TCTCCTCCTCCAGGYCTGAA-5' and for the nested PCR 5'-CCAGTGGTATCAACTCAA-3' and 3'-ATTTCTAAGTCCCCTCCTGA-5'. PCR products were sequenced clonally using the TOPO II cloning system (Invitrogen, Paisley, UK). Eleven to twenty-three clones from each subset were sequenced bi-directionally using the BigDye Terminator Cycle Sequencing kit and analyzed with the ABI 377 automated sequencer (Applied Biosystems, Foster City, CA, USA). Quality of the sequences was analyzed using CodonCode Aligner version 1.5.1, after which the sequences were aligned with BioEdit and adjusted manually with respect to the gp120 open reading frame and according to reference sequences from the Los Alamos HIV sequence database [22]. Molecular evolutionary analyses were conducted using MEGA version 4 [24]. Tamura-Nei was used as distance parameter and inter-patient cross-contamination was ruled out. Statistical analyses were performed using the Mann-Whitney test.

### Sequence data

The sequences described here were allocated the following Genbank nucleotide accession numbers: GQ389219, GQ389220, GQ389225, GQ389227 and GQ389228.

## Results

### Patient description and HIV-1 quantification in CD4^+ ^lymphocyte subsets

**Figure 1:**
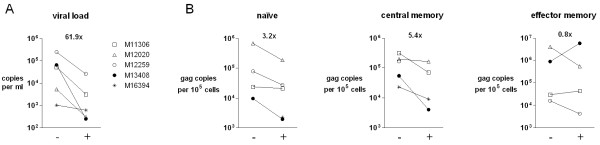
**Viral load and cellular infection levels before and after initiation of ART**. (A) Viral load values were calculated before (-) and after (+) initiation of ART and are plotted on logarithmic scale. The median decline in copy number is inserted within the graph. (B) The number of HIV-1 gag copies per 10^5 ^cells of the respective cell subset is depicted on the y-axis in logarithmic scale. An occasional subset was not included due to a large difference between the duplicate measurements.

We studied the effect of antiretroviral therapy on HIV-1 infection levels of naïve, central memory and effector memory CD4^+ ^lymphocyte populations and on the viral quasi-species present in these subsets, two to five weeks after initiation of ART. The five patients studied harbored various HIV-1 subtypes (A, C, D and F) and demonstrated a wide range of viral load values and CD4 counts (Table 1). Three out of five study subjects received an RT inhibitor (AZT), one a protease inhibitor (APV) and one received a three drug regimen (d4T/3TC/RTV). Plasma viral load declined in four individuals by 1 to 2.4 log and one subject (M16394) experienced only a small plasma load decline (Figure 1A). This patient already had a low viral load prior to therapy (1,026 copies/ml). Additionally, this patient had a high CD4 count at time of therapy initiation (800 cells/μl), which did not rise following therapy. In three of the four patients with complete data sets intracellular HIV-1 infection levels decayed by comparable levels for all cell subsets analyzed, by up to 1.1 log (Figure 1B). One exception was the effector memory population of subject M13408, the individual receiving the triple regimen, where infection levels significantly increased 6.5-fold. The drops in plasma viral loads would suggest that resistance has not occurred in the patients tested during the short time period of study.

### Influence of therapy on HIV-1 quasi-species in CD4^+ ^lymphocyte subsets

**Figure 2:**
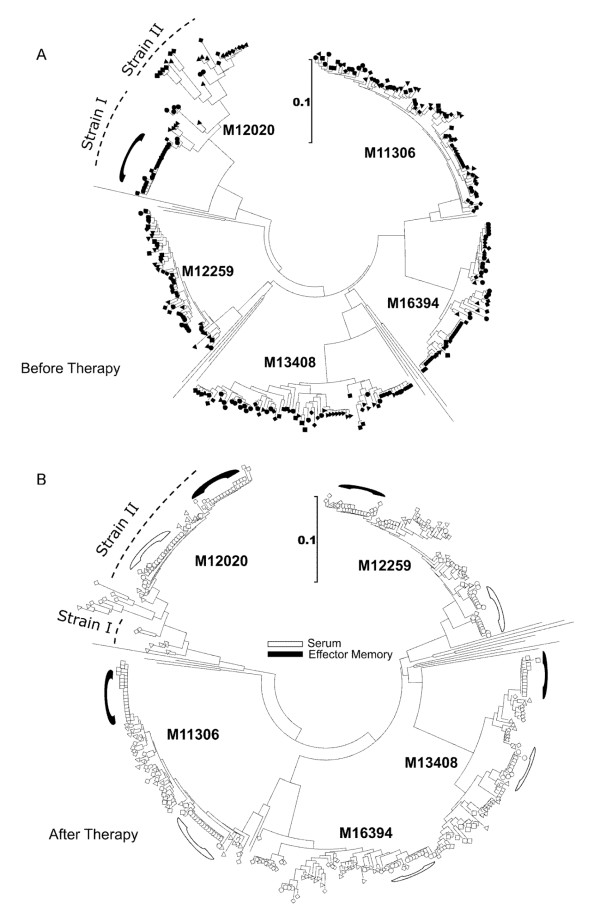
**Neighbor-joining phylogenetic analysis of the gp120 virus sequences**. The Kimura-2 parameter and 100 replicates were used to calculate nucleotide distances and sequences from the Los Alamos HIV-1 database were used as reference strains. Circles indicate sequences from serum, diamonds from naïve CD4^+ ^T cells, triangle from central memory and squares from effector memory cells. (A) Phylogeny of the strains isolated before initiation of therapy (B) Phylogeny of the strains isolated after therapy initiation. The black curved lines indicate strains from the effector memory population and the white curved lines indicate strains from serum. The dotted line indicates the two virus strains co-circulating in subject M12020.

**Figure 3:**
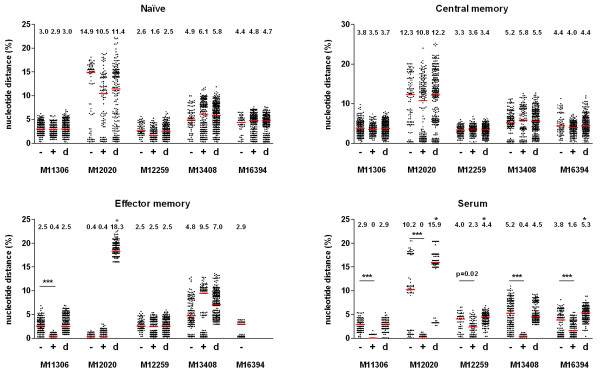
**Diversity and divergence of the viral quasi-species**. (A) From each patient pair-wise nucleotide distances before (-) and after (+) initiation of therapy were calculated for each cell subset and serum. Nucleotide distance is presented as percentage and the red bar represents the median value. Pair-wise distances between both time-points were calculated (d) and when this value was higher than the diversity of either time-point it was identified as viral divergence, indicated by an asterisk. Statistical significance was calculated for the difference in diversity before and after therapy start; *** = p < 0.0001. Data from the effector memory subset of M16394 was not available.

Our goal was to determine how therapy affected the virus variants within naïve, central memory and effector memory CD4^+ ^T cell subsets during the initial phase of therapy. Before therapy initiation, phylogenetic analysis of the C2V3 region of HIV-1 gp120 envelope did not demonstrate compartmentalization of the virus quasi-species within serum or CD4^+ ^T cell subsets (Figure 2A). Only effector memory-derived sequences from M12020 clustered. After initiation of therapy loss of diversity was observed predominantly in serum, but also within the effector memory subset (Figure 2B). Naïve- and central memory-derived virus showed modest changes in diversity. The loss of diversity was highly significant in serum (p = 0.02 for subject M12259 and p < 0.0001 for all other patients; Figure 3). No diversity loss was observed in the naïve or central memory compartments.

To measure genetic evolution of the viral quasi-species, pair-wise distances were calculated between the virus populations before and after start of therapy. In serum, divergence of the viral quasi-species was observed in three patients (indicated by an asterisk; Figure 3). This indicated selection of serum variants due to therapy introduction. Viral divergence was absent in all cell subsets, with the exception of the effector memory subset in subject M12020. The absence of changes in viral diversity and divergence within naïve and central memory subsets as opposed to effector memory cells and serum indicates that during early therapy the plasma and effector memory cell compartments are more susceptible to the effects of the drugs.

**Figure 4:**
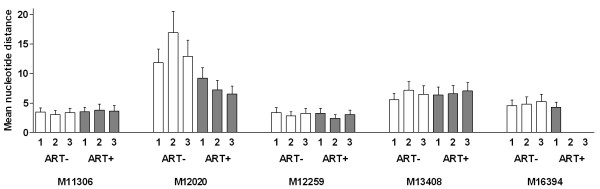
**Inter-group nucleotide diversity**. Before (ART-; white bars) and after (ART+; grey bars) therapy initiation, the mean difference in nucleotide distance was calculated using the Neighbor-joining model and the Kimura-2 parameter method. Each viral compartment was compared with all others (1: naïve - central memory, 2: naïve - effector memory and 3: central memory - effector memory).

To investigate the relatedness of virus strains among the cellular fractions the genetic distances between HIV-1 sequences derived from the various cellular fractions were calculated. Four out of five individuals demonstrated comparable distances before and after start of therapy ranging from 2.4% to 7.2% (Figure 4). After therapy initiation no change in distances were observed and were found to be similar within each of the cellular subsets. Subject M12020 was interesting since inter-subset distances before therapy were not only higher than those from all other individuals, but also higher than values observed following therapy (Figure 4). This individual was found to be infected with two different subtype D virus strains (strain I and II) as shown by phylogenetic analysis (Figure 2A). In addition, the analysis of virus sequences with DNAsp software indicated that up to 11 possible recombination breakpoints could be detected suggesting that these two virus strains were co-circulating within this individual for some time (data not shown). Before therapy, strain I was dominant in the effector cell population, while the other cell subsets harbored strain II. Both strains were present in serum. After therapy start, strain I disappeared from the effector memory subset but remained in some central memory cells (Figure 2B). The replenishment of this cell subset by a different virus strain correlated with viral divergence (Figure 3). Inter-subset virus distances approached values observed for the other patients harboring mono-infections, demonstrating that although some cell populations may be more sensitive to the effects of antiretroviral therapy, differences in sensitivity amongst virus strains also exists. These data indicate that the occurrence of dual HIV-1 infection could be an additional hurdle for therapy to succeed.

### Influence of therapy on V3 charge

**Figure 5:**
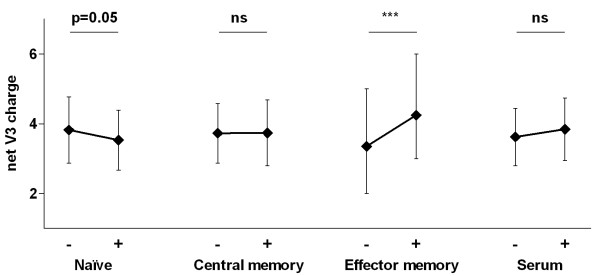
**Change in V3 charge after initiation of ART**. From all cellular subsets and serum the net V3 charge of each viral clone was calculated. The net V3 charges of all patients were grouped per time-point before (-) and after (+) initiation of ART. The graph depicts the mean value with standard deviation. *** = p < 0.0001 and ns = not significant.

Previous observations by our group and others have shown that V3 charge influences co-receptor usage [25,26]. Since CD4^+ ^lymphocyte sub-populations differ in co-receptor expression levels, we analyzed whether therapy initiation affected the V3 charge of the virus quasi-species in serum and lymphocyte subsets due to the variant expression profile. We therefore compared the V3 charge from all sequences found in the cell subsets before and after start of therapy. Sequences from all five patients were grouped together and we observed a clear increase in V3 charge within the effector memory subset in three out of four subjects (Figure 5; p < 0.0001). Within the central memory subset the V3 charge did not change whilst alterations in serum varied per patient (Figure 5; no significance). Within the naïve subset the V3 charge decreased systematically in all patients (p = 0.05), indicating that characteristics such as co-receptor usage may be involved in viral selection following initiation of therapy.

## Discussion

In our study we observed comparable viral decay within all CD4^+ ^lymphocyte sub-populations in the peripheral blood, except for one effector memory subset, confirming our previous observation that all CD4^+ ^lymphocyte subsets are productively infected with HIV-1 [9]. The results also confirm findings from other studies demonstrating comparable decay of productively infected cells in peripheral blood [21,27]. A report on preferential HIV-1 inhibition during AZT treatment in activated cells over slowly dividing cells *in vitro*, may indicate that the vast majority of virus in the circulation comes from activated cells [28]. Although naïve and central memory lymphocyte subsets contain more long-lived resting cells than the effector memory subset and outnumber this subset, no difference in viral decay was observed.

Two to five weeks after initiation of ART represents the start of the second phase of viral decay, with loss of long-lived infected cells [29]. Here we study the early effects of therapy on the virus populations found in the three different lymphocyte subsets studied and compare to the changes observed in the plasma. It may be too early to detect differences in virus composition in cell populations with a slower decay rate as may be seen at a later stage when therapy is completely suppressing virus replication. In addition, the accumulation of replication-incompetent proviral DNA in these cell subsets together with the high rate of virus production by effector memory cells may in part influence the decreased viral diversity within effector memory cells, whilst no effect was observed for the other cell subsets during the short period of the study. M13408 was the only patient who received a triple therapy regimen and who surprisingly demonstrated an increase in effector memory infection levels. Perhaps these cells possess high P-glycoprotein efflux activity decreasing intracellular antiviral drug concentrations [30]. Although blood CD4^+ ^lymphocyte levels only represent a minor fraction of the total body lymphocyte population, memory subsets in blood versus gut and lymphoid tissue counterparts are infected to the same extent [20,31], thereby indicating that studying HIV-1 infection in blood is a good representation of events that occur in other tissues. With respect to our approach of cloning prior to sequencing we argue that direct sequencing would circumvent a possible cloning bias, although neither method is more skewed than the other and both provide for a similar measure of diversity [32]. Furthermore, sequence bias can occur through preferential PCR amplification and since we do not identify this in all fractions studied or for all time-points analyzed from the same patients we feel this can be ruled out. We are confident that when we identify a restricted sequence this is representative of the viral quasi-species present within that specific fraction. In all likelihood low diversity can reflect either low infectivity or over representation of a fast replicating strain.

The more pronounced changes observed in diversity of cell-free over cell-associated virus can be explained by the difference in half-life, which can severely reduce serum copy numbers [29]. Although virus diversity in serum decreased after initiation of therapy the pair-wise distances calculated between time-points before and during therapy increased, indicating different genetic characteristics of the virus after introduction of therapy. Virus may be produced by other cell types or derived from compartments less accessible to antiretroviral drugs [19,33-35]. This is in agreement with studies demonstrating that rebound virus is distinct from variants present before start of therapy [36,37]. The absence of divergence in the cell subsets (apart from with patient M12020) can be explained by a moderate drop in infection levels and smaller changes in diversity. In M12020 the compartmentalization of effector memory-derived virus pre-therapy indicates that in the case of dual HIV-1 infection one strain preferentially infects a specific CD4^+ ^lymphocyte subset. We have previously observed in dual HIV-1 infection that one strain replicates preferentially within different cell types when compared with another strain indicating that the host cell environment influences viral replication [9,38]. The shift in balance between strains I and II is likely caused by therapy, although differences in host immune pressure, virus fitness as well as high turnover rates of the specific cell subset may also play a role. The complete and specific infection of effector memory cells by strain I and the rapid replenishment with a different virus strain indicate that this cell subset may easily facilitate infection by different variants. Although strain I was not detected in serum during therapy, its presence in long-lived central memory cells at that time-point ensures persistence of both variants. This increases the chances of recombination and therapy resistance, raising questions as to the efficacy of antiretroviral therapy in dual-infected individuals [39]. This is in line with the more resistant phenotype of HIV-2 over HIV-1 in dual-infected persons [40].

The pronounced increase in the gp120 V3 charge in effector memory cells in three out of four patients reflects increased sensitivity to therapy of virus within this cell subset. It has been speculated that such changes can influence co-receptor usage, including a possible switch towards CXCR4 usage [25,26,41,42]. Four weeks of therapy restores CCR5 expression levels, which are increased during HIV-1 infection, while CXCR4 expression levels demonstrate a modest change [43].

## Conclusions

In conclusion, ART resulted in a comparable decay of HIV-1 infection levels in naïve and central memory subsets with minor to no changes in the viral quasi-species present. HIV-1 copy numbers in the effector memory subset not always decreased and the virus in this cell subset and in serum appeared to be more sensitive to therapy. We also observed variant sensitivity among virus strains in a dual-infected individual. These results provide better insights into the viral dynamics within CD4^+ ^lymphocyte subsets during early therapy.

## Abbreviations

HIV-1: human immunodeficiency virus type 1; ART: antiretroviral therapy; PBMC: peripheral blood mononuclear cells.

## Competing interests

The authors declare that they have no competing interests.

## Authors' contributions

EJH, MJG, EB and RMvdS performed the experiments; EJH wrote the manuscript and performed statistical analyses; GP and WAP supervised and reviewed the manuscript. All authors read and approved the final manuscript.
